# Harsh Parenting and Trajectories of Emotional and Behavioural Difficulties in Autistic Children

**DOI:** 10.1007/s10803-023-06167-4

**Published:** 2023-11-04

**Authors:** Georgia Cronshaw, Emily Midouhas

**Affiliations:** Department of Psychology and Human Development, IOE, UCL′s Faculty of Education and Society, London, UK

**Keywords:** Autism spectrum condition, Emotional and behavioural difficulties, Harsh parenting, Trajectories, Early-to-middle childhood

## Abstract

Autistic children show higher rates of co-occurring emotional and behavioural difficulties compared to other children in the general population. However, the environmental factors which contribute to the development of emotional (internalising) and behavioural (externalising) difficulties in autistic individuals are poorly understood. This study sought to investigate the association between harsh parenting (smacking, shouting, telling off) and the trajectories of emotional and behavioural difficulties in autistic children from ages 3 to 7. A sample comprising of 349 autistic children participated from the UK’s Millennium Cohort Study. Associations between harsh parenting and child emotional and behavioural difficulties were modelled using multilevel growth curve models. In autistic children, harsh parenting was associated with total emotional and behavioural difficulties and behavioural (but not emotional) problems concurrently. Moreover, harsh parenting was not associated with changes in emotional and behavioural difficulties over time. Harsh parenting may have an important role in externalising problems in young autistic children indicating the need for public health strategies which educate parents on its effects.

**Supplementary Information:**

The online version contains supplementary material available at 10.1007/s10803-023-06167-4.

## Introduction

Autism spectrum condition is a neurodevelopmental condition that is characterised by challenges with both verbal and non-verbal social interactions and repetitive behaviours (Atherton et al. 2022). It has a global prevalence of around 1–2% (Zeidan et al. 2022). Autistic individuals are at risk of having co-occurring mental health conditions (Lai et al. 2019); for example, one study showed that around 70% of autistic children meet the diagnostic criteria for at least one psychiatric condition (Simonoff et al. 2008). Emotional (internalising) problems, such as being anxious, withdrawn or depressed and behavioural (externalising) problems, including being disruptive, hyperactive and aggressive, have been shown to be experienced at elevated rates in autistic children (Mandy et al. 2022)  and have been linked with later mental health issues (Hammer et al. 2017; Simonoff et al. 2012). An important area of investigation is the contribution of the environment (e.g., home, school, neighbourhood) for the development of such co-occurring difficulties as a potential target for intervention.

Parents and parenting practices play an important role in the onset and maintenance of children’s mental health but research into the parenting environment for autistic children’s outcomes is limited. This may be due to a historical misunderstanding that autism was caused by “cold’’ parenting (Evans, 2013), making it a controversial topic of exploration and discussion. However, given the elevated rates of emotional and behavioural difficulties, parents and other carers of autistic children are often faced with unique challenges and burdens (Weir et al. 2020) that may shape how they approach parenting. Stress associated with parenting autistic children may put parents at risk of psychological distress (Estes et al. 2013) which is linked to harsher parenting (Shawler & Sullivan 2017). Harsh parenting refers to coercive acts and negative emotional expressions that parents direct towards their child including both verbal aggression (e.g., shouting or telling off) and physical aggression (e.g., smacking or spanking), conceptualised along a continuum of parenting, with child maltreatment at the extreme end (Gershoff, 2002). It is associated with risk of emotional and behavioural difficulties, both in the short-term and long-term (Jaffee et al. 2004). Conversely, a parenting environment where there is sensitivity, warmth and consistency is seen as promotive of children’s emotional and behavioural adjustment.

In general population samples, harsh parenting has been associated with conduct, aggression, hyperactivity and inattentiveness problems (Bender et al. 2007; Taylor et al. 2010; Vostanis et al. 2006) and, in fewer studies, it has also been associated with emotional problems, including anxiety and depression (Hecker et al. 2016; Rajyaguru et al. 2019). However, transactional models of development suggest that parents and children mutually influence each other. Coercion theory (Patterson, 2002) suggests that parent–child interactions can lead to coercive dynamics, where both parent and child may reinforce behavioural problems, resulting in increased difficulties. Bidirectional associations have indeed been found between harsh parenting and behavioural problems in typically developing children (Speyer et al. 2022) where both parents’ and children’s behaviours mutually reinforce each other. We would also expect these dynamics to operate for autistic children and their parents as well.

A small body of literature (Bader & Barry, 2014; Dieleman et al.^2017^ ; Greenberg et al. 2006; Lin et al. 2023; Lindsey et al. 2020; Maljaars et al. 2014; McRae et al. 2018) has examined the relationship between harsh parenting practices and emotional and behavioural outcomes in autistic children, finding harsh parenting to be negatively related to behavioural problems and, to a lesser extent, to emotional problems in autistic children. However, most studies used cross-sectional data, finding associations between harsh parenting and externalising problems (Lin et al. 2023; Maljaars et al. 2014; McRae et al. 2018). One cross-sectional study also found a relationship with internalising problems (McRae et al. 2018) and in another study similar results were observed longitudinally (Lindsey et al. 2020). A notable study of the reciprocal associations between negative controlling parenting (capturing discipline and harsh punishment) and child adjustment problems, in a sample of 139 autistic children over a 9-year period, found bidirectional associations with externalising problems but only parent effects on internalising problems (Dieleman et al. 2017); Greenberg et al. (2006) observed similar bidirectional effects, while Bader and Barry (2014) found a parent (criticism/hostility) effect only on externalising problems.

Taken together, few studies have examined the longitudinal relationship between harsh parenting and emotional and behavioural trajectories in autistic children, helping us to understand how harsh parenting may relate to changes in problems as children grow older. Moreover, investigating the potential impact of harsh parenting for autistic children’s adjustment can also contribute to our broader understanding of the parenting environment and its role in the development of emotional and behavioural difficulties in autistic children, to inform the identification of effective parenting interventions for these problems.

In the present study, we used data from a U.K. general population cohort of families with young children. We investigated the relationship between maternal harsh parenting, defined here as smacking, shouting, and telling off, and autistic children’s emotional and behavioural trajectories from ages 3 to 7. We examined, in autistic children, whether maternal harsh parenting was related to trajectories of total difficulties (both emotional and behavioural together) as well as emotional and behavioural difficulties across ages 3 to 7, adjusting for potential confounders. We hypothesised that children exposed to maternal harsh parenting would have - concurrently and longitudinally - more emotional and behavioural difficulties relative to children without this exposure.

## Methods

### Data and Participants

We used data from the Millennium Cohort Study (MCS), a national birth cohort study that follows the lives of around 19,000 children, and their families, born across England, Scotland, Wales and Northern Ireland from the years 2000–2002. Children’s eligibility was identified using the U.K. government child benefit records, as it has near universal coverage (Connelly & Platt, 2014). At the time of this study, eight survey sweeps had been conducted, starting at age 9 months with follow ups at ages 3, 5, 7, 11, 14, 17 and 22 years. The MCS is housed at the Centre for Longitudinal Studies at the UCL Institute of Education, and all data are freely accessible through the UK Data Service. The MCS received ethical approval from the National Health Service Research Ethics Committee, with ethical approval being sought for all MCS follow-up surveys. Additional ethical approval was granted for this study from the UCL Institute of Education.

**Fig. 1:**
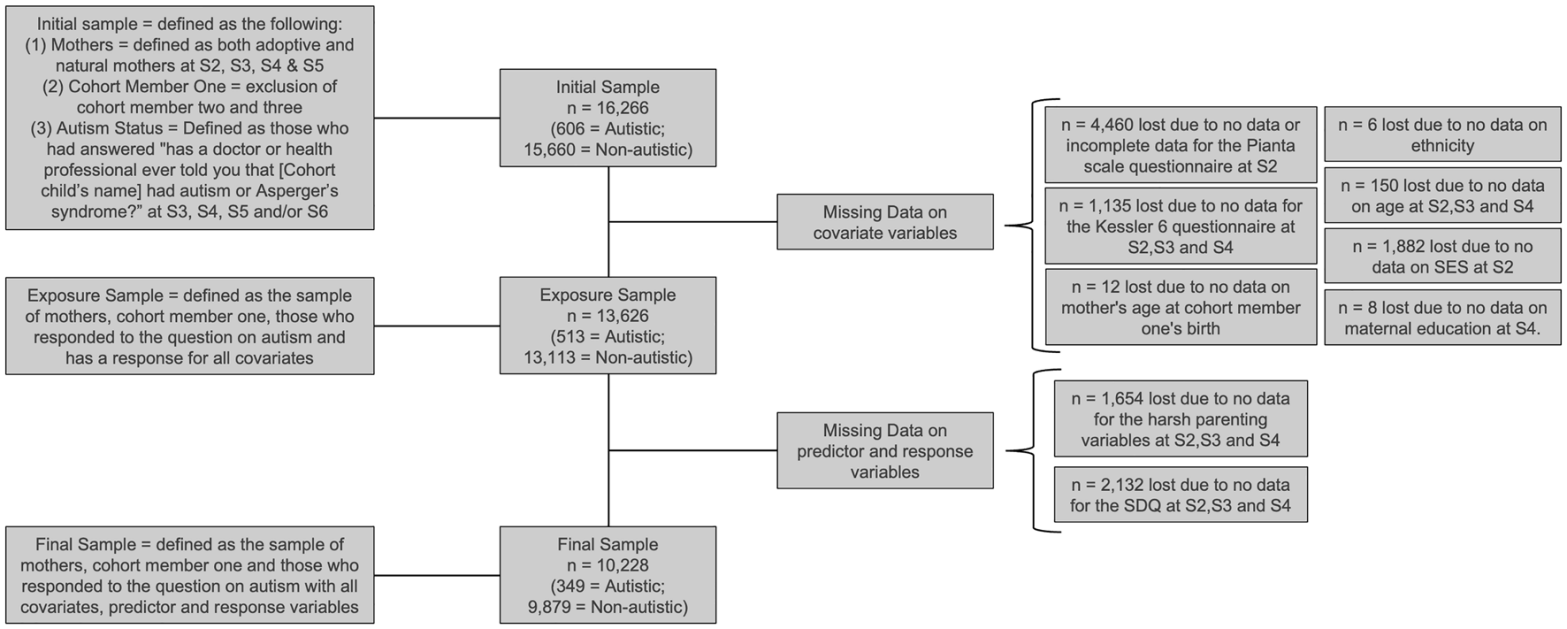
*Sample selection process*. Missing data does not add up to the difference between (1) initial sample and exposure sample or (2) exposure sample and final sample as there is overlap in the individuals missing

Data from sweeps 1 (age 9 months) to 6 (age 14 years) were used in this study. Figure 1 outlines the study’s sample selection process. In sweep 1, 18,522 families participated, whereas in sweeps 2, 3, 4, 5 and 6 there were 15,590, 15,246, 13,857, 13,287 and 11,872 participating families respectively. The initial sample consisted of singletons and the first-born of twins/triplets, with mothers (both natural and adoptive) as the main respondent. To be included in the autistic sample (n = 349), a child had to have a response from any eligible respondent for the question: “Has a doctor or health professional ever told you that [Cohort child’s name] had autism or Asperger’s syndrome?”  at least once from sweeps 3 to 6. This approach was used to address the possibility of delayed autism diagnoses, particularly among autistic girls.  Those who answered “no” at all sweeps with valid data were considered non-autistic (n = 9879). The MCS provided weights and stratifying and clustering variables to account for attrition and the complex sampling design (Plewis, 2007). Individuals with missing data for non-repeated measures were not included in the sample; however, for time-varying measures, cases with at least one valid data point were included. We did not need to use complete-case analysis on repeated measures as growth curve models are able to use unbalanced data (Grimm, Ram & Hamagami, 2011).

Within the autistic sample (n = 349), 22.1% were female and 77.9% were male; this is close to the 1:3 ratio expected in the general population (Loomes et al. 2017).

### Measures

#### Harsh Parenting

Harsh parenting, defined as coercive acts and negative emotional expressions that parents direct towards their child (Gershoff, 2002), was measured with three items assessing how often the parent smacks, shouts at and tells off their child when the child misbehaves. These items were taken from the Parent–Child Conflict Tactics Scale (CTS; Straus et al. 1998) measured at sweeps 2, 3 and 4. The CTS is a 7-item measure capturing the amount of negative parental conflict tactics, including both emotional and physical tactics used when the child misbehaves (Straus et al. 1998). Items were scored with a frequency scale ranging 0 (never) to 4 (daily). A continuous variable was produced to reflect harsh parenting, summing together the items. These items capture the physical punishment and verbal aggression aspects of harsh parenting and have been previously measured in cohort studies including the MCS in this way (Flouri & Midouhas, 2017; Rajyaguru et al. 2019; Speyer et al. 2022). The harsh parenting score ranged from 0 to 12. Internal consistency ranged from α = 0.65 to 0.67, which is considered adequate (Tavakol and Dennick 2011).

#### Child Emotional and Behavioural Difficulties

Emotional and behavioural difficulties were measured at ages 3, 5 and 7 with the parent-reported Strengths and Difficulties Questionnaire (SDQ) (Goodman, 1997; Ortuño-Sierra et al 2015). The SDQ has been shown to reliably measure externalising and internalising problems in children (Mieloo et al. 2012), but has also been shown to act as a strong predictor of mental health problems in autistic individuals (Simonoff et al. 2012) and used within other longitudinal studies looking at emotional and behavioural difficulties within this population (Baird et al. 2023; Flouri et al. 2015; Mandy et al. 2022; Midouhas et al. 2013). The SDQ has 25 items on psychological attributes, uses a three-point scale ranging 0 (not true), to 2 (certainly true), and can be split into five domains: (1) Emotional Symptoms; (2) Conduct Problems; (3) Hyperactivity/Inattention; (4) Peer Problems; and (5) Prosocial Behaviour. To create the variables, we summed: (1) 1 to 4 to generate a total difficulties score; (2) 1 and 4 to create an internalising problems score and (3) 2 and 3 to create an externalising problems score. A total-difficulties score of greater than or equal to 17 is considered ‘abnormal’ and therefore clinically relevant, and a score at or below 13 is considered to be in the normal range of difficulties (Bryant et al. 2020; Fleitlich et al. 2000). Internal consistency across ages 3, 5 and 7 were: (1) total difficulties (α = 0.76 to 0.82); (2) internalising problems (α = 0.53 to 0.69) and (3) externalising problems (α = 0.77 to 0.80).

#### Covariates

In order to get closer to isolating the association between harsh parenting and child difficulties, we adjusted for several possible confounding factors: (1) Maternal mental health was measured using the Kessler 6 Psychological Distress Scale (Kessler et al. 2002) at child ages 3, 5 and 7, a 6-item scale of non-specific psychological distress with responses ranging from 0 to 24. We used a total score (α = 0.85 to 0.87 across ages 3 to 7). Mother’s mental health is evidenced to predict both discipline practices use and child behavioural difficulties (Gadermann et al. 2012); (2) Highest maternal educational qualification achieved by age 7 was also measured as either university educated or not university educated. Maternal education level has also been shown to predict both parenting and the behavioural outcomes of children in later life (Awada & Shelleby, 2021); (3) Poverty status was defined as whether the family lived below the poverty line, defined as 60% of the median income in the UK and has been shown to influence both harsh parenting and child difficulties (Kivimäki et al. 2020); (4) Mother’s age at child’s birth was also adjusted for; mother’s age at birth has been shown to be related to parenting practices and subsequent child behavioural outcomes (Coyne et al. 2013). Biological sex, child’s age in years and ethnicity (white and other) were also adjusted for.

To adjust for the bidirectional relationship between parent and child behaviours, parent–child relationship at age 3 was measured with the Child-Parent Relationship Scale (Pianta: Short Form; CPRS) which measures the mother’s report of the child’s relationship with her mother. The items capture the mother’s feelings and beliefs about her relationship with her child, and about the child’s behaviour toward the mother. The CPRS is a 15-item scale with a five-point frequency scale ranging from 1 (Definitely does not apply) to 5 (Definitely applies) (α = 0.77). We created a continuous total score by summing all item responses (Driscoll & Pianta, 2011).

### Statistical Analysis

Initial descriptive statistics were conducted on the sample using SPSS (version 29). Regression analyses and correlations were conducted in R v. 4.2.1 (R Core Team, 2022) using the lme4 package for growth curve models. Analyses used weights provided by the MCS to account for the study design and attrition.

Initially, correlations (product moment correlations coefficient) were inspected between the predictor variable, harsh parenting, and the different response variables that indicate psychopathologies: (1)​​ total difficulties; (2) internalising problems; and (3) externalising problems.

**Fig. 2:**
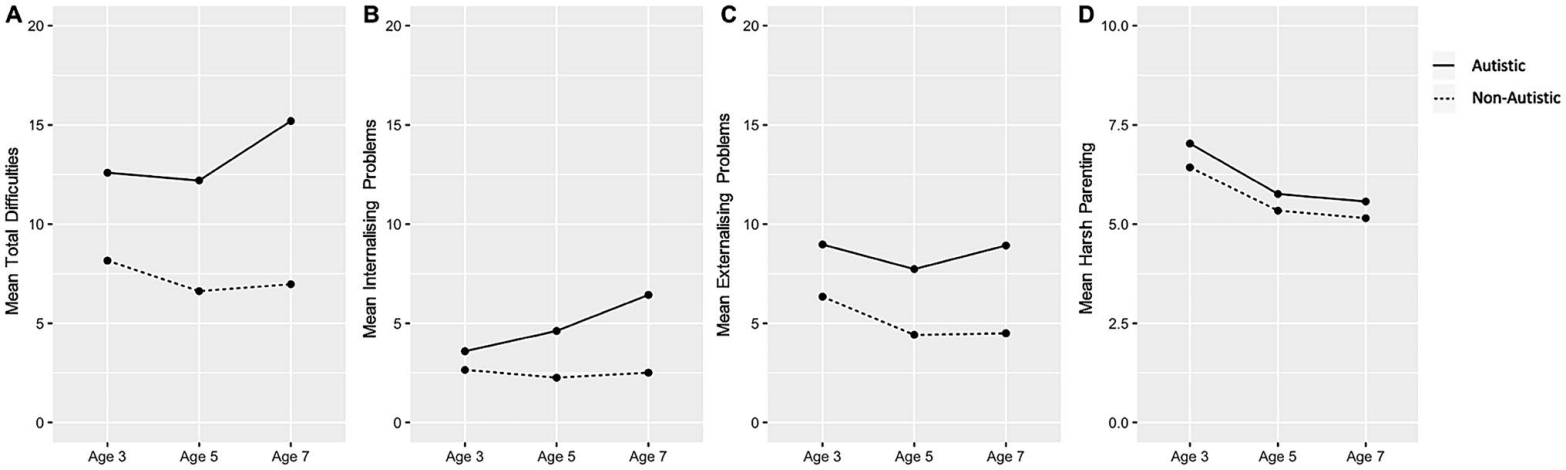
*Weighted mean trajectories of emotional and behavioural difficulties*. **a** Total difficulties **b** Internalising problems **c** Externalising problems and **d** harsh parenting score from ages 3 to 7 in autistic and non-autistic children

Then, trajectories of difficulties were assessed using growth curve models (Supporting Information: Table S1). The structure of the MCS data is consistent with a hierarchical structure given the use of longitudinal repeated measures; thus, to avoid underestimation of standard errors, two-level growth curve models were utilised with level one being occasion and level two being the child. We then modelled the role of harsh parenting on individual trajectories of difficulties: (1)​​ total difficulties; (2) internalising problems and (3) externalising problems. To capture individual trajectories, a random slope for child’s age in years was modelled, enabling individual total difficulties, internalising problems, and externalising problems to vary across time. When adding random effects, “maximal” random effects structure was used (Barr et al. 2013). We also centred age at the grand mean (5.13 years) and used age-squared to assess for a non-linear relationship between age and each difficulty domain (as weighted average trajectories for children followed a U-shaped trajectory; Fig.2). As such, the models estimated both the random and fixed linear trajectories of all domain scores and the fixed non-linear trajectories (with only three timepoints, modelling a random effect for age-squared was not possible) from ages 3 to 7 years. Due to the use of centred age, all the main effects (not interacted with age) predicted total, internalising and externalising difficulties at age five. Given the number of children in the sample, we used maximum likelihood estimation in all models, as the bias in this method is negligible for the numbers used (Kwok et al. 2008). For all non-repeated measures, complete-case analysis was used. We adjusted for the strata in all regression models: England advantaged, England disadvantaged, England ethnic, Scotland advantaged, Scotland disadvantaged, Wales advantaged, Wales disadvantaged, Northern Ireland advantaged and Northern Ireland disadvantaged.

To understand if there are differences between the autistic sample and non-autistic sample, we conducted a descriptive comparative analysis. We also conducted a supplementary analysis of the growth curve models in the non-autistic sample.

## Results

### Descriptive Results

 Autistic children exhibited on average higher rates of total difficulties compared to non-autistic children across all ages (Fig. 2a). This was also the case for both internalising and externalising problems (Fig. 2b and c). Figure 2 shows the weighted mean scores for all problem types and harsh parenting. The autistic sample showed increases in total difficulties, internalising and externalising problems across time, whereas the non-autistic group showed decreases. Moreover, harsh parenting was used at higher levels in both autistic and non-autistic children at age 3 compared to ages 5 and 7; however, parents reported comparable levels of harsh parenting for both autistic and non-autistic children at each time point (Fig. 2d). Tables 1 and 2 show descriptive statistics for continuous and categorical variables respectively. The autistic sample had lower levels of maternal warmth than that of the non-autistic sample; moreover, mean maternal age at birth was slightly lower for the autistic sample. Both autistic and non-autistic samples showed similar proportions of mothers who were university educated; however, a higher proportion of families were below the poverty line in the autistic sample compared to the non-autistic sample.

**Table 1:** Summary table of weighted continuous variables

Characteristics	Total				Autistic				Non-Autistic			
	min	max	mean	*SD*	min	max	mean	*SD*	min	max	mean	*SD*
Age 3 (Sweep 2)												
Age (years)	2.65	4.56	3.13	0.19	2.94	4.20	3.14	0.22	2.65	4.56	3.13	0.19
Parent–Child Warmth	15.0	60.0	49.2	6.50	23.0	60.0	45.71	7.84	15.0	60.0	49.35	6.71
Maternal Age at CM Birth	15.0	46.0	29.53	5.59	17.0	40.0	27.47	5.48	15.0	46.0	29.59	5.59
Maternal Mental Health	0.00	24.0	20.8	6.79	1.00	24.0	19.3	4.76	0.00	24.0	20.9	3.54
Harsh Parenting	0.00	12.0	6.36	2.21	1.00	12.0	7.03	2.38	0.00	12.0	6.43	2.31
Total Difficulties	0.00	30.0	9.04	4.74	2.00	29.0	12.59	5.96	0.00	30.0	8.16	4.53
Internalising Problems	0.00	18.0	2.68	2.18	0.00	13.0	3.59	2.70	0.00	18.0	2.65	2.15
Externalising Problems	0.00	20.0	6.43	3.61	1.00	20.0	8.97	4.24	0.00	20.0	6.34	3.55
Age 5 (Sweep 3)												
Age (years)	4.40	6.13	5.20	0.24	4.64	5.86	5.18	0.25	4.40	6.13	5.20	0.24
Maternal Mental Health	0.00	24.0	21.0	3.61	0.00	24.0	19.30	4.69	3.00	24.0	21.1	3.55
H arsh Parenting	0.00	12.0	5.44	1.86	0.00	12.0	5.76	2.03	0.00	12.0	5.43	1.86
Total Difficulties	0.00	34.0	6.82	4.50	0.00	32.0	12.20	6.70	0.00	34.0	6.62	4.28
Internalising Problems	0.00	17.0	2.35	2.26	0.00	17.0	4.62	3.70	0.00	16.0	2.26	2.15
Externalising Problems	0.00	20.0	4.45	3.23	0.00	18.0	7.73	4.22	0.00	20.0	4.42	3.12
Age 7 (Sweep 4)												
Age (years)	6.37	8.15	7.22	0.24	6.62	7.82	7.21	0.25	6.37	8.15	7.22	0.24
Maternal Mental Health	0.00	24.0	21.0	3.74	3.00	24.0	19.0	4.65	0.00	24.0	21.0	3.68
Harsh Parenting	0.00	12.0	5.17	1.85	1.00	11.0	5.57	2.00	0.00	12.0	5.15	1.85
Total Difficulties	0.00	37.0	7.29	5.20	0.00	35.0	15.2	7.85	0.00	37.0	6.97	4.80
Internalising Problems	0.00	18.0	2.66	2.62	0.00	18.0	6.43	4.35	0.00	17.0	2.51	2.41
Externalising Problems	0.00	20.0	4.67	3.54	0.00	19.0	8.92	4.69	0.00	20.0	4.50	3.38

*max*  maximum value in the specific sample; *min*  minimum value in the specific sample *SD*  Standard deviation

**Table 2:** Summary table of categorical variables

Characteristics	Total (n = 10,228)		Autistic (n = 349)		Non-autistic (n = 9,879)	
	n	%	n	%	n	%
Sex						
Male	5207	50.9	272	77.9	4935	50.0
Female	5021	49.1	77	22.1	4944	50.0
Ethnicity						
White	9270	90.6	330	94.6	8940	90.5
Other	958	9.4	19	5.6	939	9.5
OECD poverty status						
Below poverty line	2832	27.7	118	33.8	2714	27.5
Above poverty line	7396	72.3	231	66.2	7165	72.5
Mother’s education						
University educated	2299	22.5	74	21.2	2225	22.5
Not University educated	7929	77.5	275	78.8	7654	77.5

*n*  number *%* percentage

Harsh parenting was significantly and positively correlated with all response variables: (1) Total difficulties; (2) internalising problems; and (3) externalising problems, with the strongest correlation with externalising problems (0.37) and the weakest with internalising problems (0.07; Table 3).

**Table 3:** Summary of correlation coefficients obtained for ages 3, 5 and 7 (n = 349)

Characteristics	Age 3 (Sweep 2)					Age 5 (Sweep 3)					Age 7 (Sweep 4)			
	1.	2.	3.	4.		1.	2.	3.	4.		1.	2.	3.	4.
Harsh parenting														
Total difficulties	0.29**					0.29**					0.31**			
Externalising problems	0.35**	0.90**				0.34**	0.88**				0.37**	0.89**		
Internalising problems	0.07**	71**	0.33**			0.10**	0.75**	0.34			0.12**	0.78**	0.41**	

*p < .05 **p < .01

### Growth Curve Regression Models

To model the relationship between harsh parenting and trajectories of difficulties in autistic children, multilevel growth curve models were used.

#### Total Difficulties

The non-linear age effect was statistically significant (β = 0.270, SE = 0.066, p < 0.001; Table 4). The main effect of harsh parenting on autistic children’s total difficulties at central age was statistically significant; here results showed that, on average, a one unit increase in harsh parenting was associated with around a half unit increase in total difficulties when holding all other predictors constant (β = 0.511, SE = 0.101, p < 0.001; Table 4). Harsh parenting was not related to the rate of change across time for total difficulties, as the interaction term for age and harsh parenting was not statistically significant. With regard to the random effects, between-child variance was higher than within–child variance, indicating stability within children; the larger variance between children may be due to differences in child characteristics (e.g., cognition) and unmeasured family factors (e.g., home environment).

**Table 4:** Fixed and random effects estimates predicting total difficulties, internalising, and externalising problems (n = 349)

			Total Difficulties			Internalising Problems			Externalising Problems		
			β	*SE*	*CI (95%)*	β	*SE*	*CI (95%)*	β	*SE*	*CI (95%)*
Fixed effects											
Constant			28.520***	2.293	[28.010–33.022]	10.519***	1.310	[7.930–13.020]	18.984***	1.415	[16.178–21.790]
Harsh parenting			0.511***	0.101	[0.312–0.709]	0.058	0.059	[− 0.58–0.174]	0.380***	0.060	[0.261–0.499]
Harsh parenting X Age			0.048	0.048	[− 0.046–0.142]	0.019	0.028	[− 0.036–0.073]	0.001	0.028	[− 0.056–0.057]
Age			0.432	0.304	[− 0.166–1.032]	0.513***	0.174	[0.171–0.855]	0.070	0.182	[− 0.290–0.431]
Age^2^			0.270***	0.066	[0.142–0.340]	0.072	0.041	[− 0.008–0.152]	0.168***	0.036	[0.096–0.239]
Female			− 0.973	0.591	[− 2.156–0.208]	− 0.452	0.331	[− 1.107–0.202]	− 0.667	0.372	[− 1.410–0.077]
Poverty status (Ref: advantaged)											
Poverty status disadvantaged			0.749	0.571	[-0.379–1.871]	0.288	0.322	[− 0.349–0.921]	0.521	0.356	[− 0.182–1.221]
University educated			− 0.967	0.604	[− 2.165–0.230]	− 0.314	0.340	[− 0.985–0.357]	− 0.830*	0.378	[− 1.578–0.082]
Ethnicity other (Ref: White)			0.363	1.191	[− 1.980–2.702]	− 0.179	0.645	[− 1.446–1.090]	0.259	0.754	[− 1.222–1.739]
Area stratum (Ref: England-advantaged)											
England disadvantaged			− 0.717	0.620	[− 1.948–0.511]	0.177	0.350	[− 0.514–0.868]	− 0.895*	0.388	[− 1.663–0.128]
England ethnic			− 0.566	1.206	[− 2.941–1.804]	0.028	0.683	[− 1.316–1.370]	− 0.217	0.760	[− 1.171–1.277]
Scotland advantaged			0.996	1.058	[– 1.093–3.079]	1.066	0.593	[− 0.099–2.235]	0.083	0.676	[− 1.255–1.418]
Scotland disadvantaged			− 0.638	1.049	[– 2.71–1.432]	− 0.236	0.590	[− 1.398–0.924]	− 0.263	0.663	[− 1.572–1.046]
Wales advantaged			2.805	1.630	[− 0.349–6.010]	1.138	0.915	[− 0.666–2.937]	1.234	0.989	[− 0.710–3.180]
Wales disadvantaged			− 0.150	0.876	[− 1.873–1.572]	0.272	0.487	[− 0.686–1.228]	− 0.467	0.555	[− 1.559–0.623]
Northern Ireland advantaged			–2.102	1.390	[– 4.850–0.645]	– 0.227	0.773	[– 1.753–1.298]	– 1.781*	0.875	[− 3.507–0.054]
Northern Ireland disadvantaged			− 0.779	1.229	[− 3.195–1.637]	− 0.253	0.683	[− 1.596–1.090]	− 0.502	0.752	[− 1.981–0.974]
Maternal warmth			− 0.309***	0.034	[− 0.376–0.241]	− 0.103***	0.019	[− 0.140–0.065]	− 0.217***	0.021	[− 0.260– 0.175]
Maternal mental health			− 0.283***	0.043	[− 0.369–00.197]	− 0.152***	0.025	[− 0202–0.101]	− 0.120***	0.026	[− 0.171–0.069]
Maternal age at birth			0.031	0.048	[− 0.064–0.125]	0.048	0.027	[− 0.005–0.101]	− 0.021	0.030	[− 0.079–0.038]
Random effects											
Level 2 (child)											
Between-child intercept variance			15.613			4.883			6.393		
Between-child slope variance			1.148			0.247			0.574		
Between-child intercept/slope variance covariance			2.638			0.992			0.834		
Level 1 (occasion)											
Residual variance			11.268			4.935			4.011		

*β* unstandardised coefficients *SE*  Standard Error *CI*  95% confidence intervals

* p  < .05 ** p  < .01 *** p  < .001

#### Externalising Problems

The rate of change in externalising problems was also non-linear (*β* = 0.168, *SE* = 0.036, *p* < .001; Table 4), with the main effect of harsh parenting being significantly associated with externalising problems at age 5 (*β* = 0.380, *SE* = 0.060, *p* < .001; Table 4). Here, a one unit increase in harsh parenting was associated with a 0.38 increase in the externalising problems score. Harsh parenting was not related to the linear rate of change in externalising problems.

#### Internalising Problems

At centred age, harsh parenting did not predict internalising problems (*β* = 0.058, *SE* = 0.059, *p* > .05; Table 4). While both externalising and total difficulties followed a non-linear trajectory, internalising problems followed a linear path (*β* = 0.513, *SE* = 0.174, *p* < .001; Table 4). However, harsh parenting was not related to this linear rate of change.

**Fig. 3:**
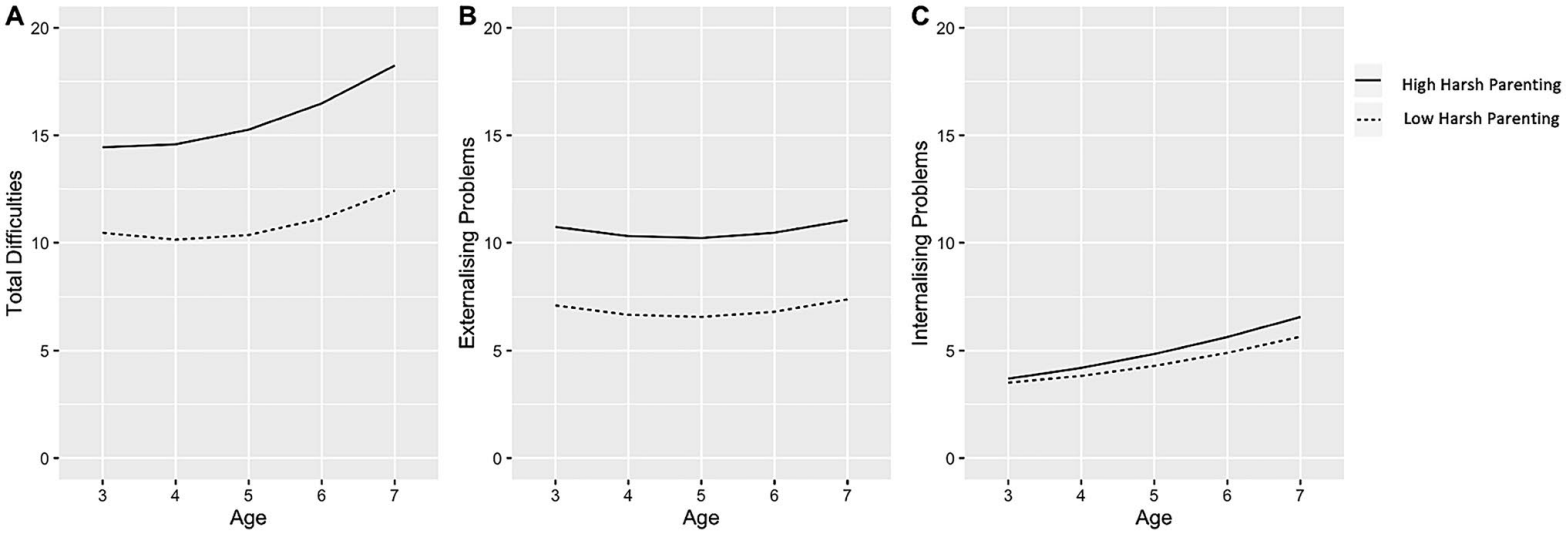
*Predicted trajectories of emotional and behavioural difficulties (n = 349)*. **a** total difficulties **b** externalising problems and **c** internalising problems from ages 3 to 7 in autistic children for both high and low levels of harsh parenting. High and low harsh parenting were calculated as the 90th and 10th percentiles respectively, with reference groups being used for categorical variables and the mean being used for continuous variables

To illustrate the results, Fig. 3a and b, and 3c show the predicted trajectories for (1) total difficulties; (2) internalising problems; and (3) externalising problems for autistic children exposed to high and low levels of harsh parenting. Figure 3a and b show the U-shaped trajectories of total difficulties and externalising problems, with the child exposed to high harsh parenting showing more problems but following a parallel trajectory to the child exposed to low harsh parenting (1 standard deviation below the mean). Moreover, Fig. 3c shows the positive linear shape of the trajectory of internalising problems; however, the gap in internalising problems between the child exposed to high and low levels of harsh parenting appears to be minimal. Notably, at a high level of harsh parenting, the autistic individual’s trajectory of total difficulties increased from “borderline” at age 3 (14.40) to an “abnormal” level by age 7 (18.24). At the low level of harsh parenting, the trajectory of total difficulties also increased, but remained “normal” moving from 10.46 at age 3 to 12.42 at age 7. The trajectory of externalizing problems varied between low and high levels of harsh parenting, demonstrating noticeable differences. The scores at both high and low levels of harsh parenting dipped at age 5 but at age 7 scores returned to a similar level as at age 3. On the other hand, internalizing problems displayed a consistent increase from age 3 to age 7, irrespective of high or low level of harsh parenting. Nevertheless, the difference between the high and low level of harsh parenting was not noticeable.

### Supplementary Analysis

As a supplementary analysis, we went on to model the relationship between harsh parenting and (1) conduct; (2) hyperactivity, (3) emotional and (4) peer problems to determine what may be driving the relationships observed in externalising problems and internalising problems. Our findings revealed statistically significant associations between harsh parenting and both conduct and hyperactivity. However, we did not observe any statistically significant relationship between harsh parenting and either peer problems or emotional problems.

Moreover, we ran these models in the non-autistic children sample. Harsh parenting was associated with all three problem types at centred age (Table S2). Moreover, there was a significant interaction term between harsh parenting and age for all three problem types. When considering trajectories between autistic and non-autistic children for high and low levels of harsh parenting, both autistic and non-autistic children showed non-linear rates of change over time for total difficulties and externalising problems and both groups showed linear changes for internalising problems (Fig S1). While autistic children showed increases in total difficulties over time at both high and low levels of harsh parenting, non-autistics did not show the same pattern of change across time; instead, they showed decreases in total difficulties.

## Discussion

Harsh parenting, the use of verbal and physical aggressive acts and emotional expressions by parents towards their children, has been studied in great depth within typically developing child samples, but we know little about the role of harsh parenting in autistic children’s co-occurring adjustment problems. Our study examined the role of harsh parenting in the trajectories of emotional and behavioural difficulties in autistic children in a general population study. We found that harsh parenting relates to behavioural difficulties in young autistic children. On average, autistic children had higher scores in broad emotional and behavioural difficulties which increased from ages 3 to 7 compared to non-autistic children. This finding could indicate that compared to the general population, autistic children may have higher rates of co-occurring psychiatric problems (Maljaars et al. 2014; Midouhas et al. 2013; Simonoff et al. 2008). Comparable amounts of harsh parenting appeared to be used by mothers of autistic and non-autistic children, consistent with Maljaars et al. (2014). Interestingly, given autistic children exhibited, on average, higher levels of behavioural (externalising) difficulties compared to non-autistic children, it could be inferred that mothers of autistic children are less likely to use harsh parenting relative to exhibited behavioural difficulties, indicating that mothers of autistic children are less likely to use harsh parenting practices at home. However, it could suggest that mothers of autistic children interpret behaviours differently and thus do not see the need for harsher forms of parenting (Reese et al. 2005). It is also crucial to emphasise, however, that interpretations of these comparisons should be considered with caution as these comparisons are not statistically based; thus, further research is needed to explore this.

As expected, harsh parenting was positively associated with both total difficulties and externalising problems in autistic children, with externalising problems likely driving the association found with total difficulties. The relationship between harsh parenting and externalising behaviours has been replicated within typically developing samples (Lansford et al. 2011; Speyer et al. 2022; Vostanis et al. 2006). This is also reflected in similar work within the autistic population (Baker et al. 2020; Lindsey et al. 2020; Maljaars et al. 2014). In our supplementary analysis, a comparable association was also observed in the non-autistic sample, but at a smaller magnitude. While making comparisons with other studies is not straightforward, as there are a range of definitions of harsh parenting, our study provides much needed longitudinal evidence that harsh parenting is positively associated with externalising problems in autistic children, suggesting that harsh parenting my influence the development of behavioural problems in autistic children.

Notably, at high levels of harsh parenting, autistic individuals’ trajectories of total difficulties (both emotional and behavioural difficulties) increased from “borderline” at age 3, to an “abnormal” level by age 7. Yet under low levels of harsh parenting, all scores remained in the “normal” range, indicating the risk of clinically relevant mental health problems in those who experience high levels of harsh parenting (Bryant et al. 2020). The scores of externalising problems varied between low and high levels of harsh parenting, with high levels of harsh parenting resulting in higher scores of externalising problems, further emphasising the potential impact harsh parenting has on externalising problems (Baker et al. 2020; Chang et al. 2003; McRae et al. 2018; Pinquart, 2017). Yet this was not seen for internalising problems.

Coercion theory proposes a potential mechanism through which externalising problems may develop in children (Patterson, 2002). It is possible that autistic children with behavioural problems also elicit harsh parenting from their parents, as shown in general population samples (Speyer et al. 2022). Harsh parenting might reinforce rather than simply increase problem behaviours, leading to elevated externalising behaviours. This is shown in autistic populations by Lucyshyn et al. (2004) who undertook an observational study and by (Dieleman et al. 2017) who looked longitudinally at the bidirectional relationship between parenting and maladaptive behaviours in autistic children. In addition, emotional regulation challenges have also been suggested as a mechanism through which harsh parenting may lead to externalising problems (Chang et al. 2003; Goagoses et al. 2022). While positive parenting acts to teach and improve a child’s self-control and behaviour through improving emotional regulation capacity (Goagoses et al. 2022), harsh parenting usually does not facilitate this and has been suggested to result in worse emotional regulation capabilities in children (Chang et al. 2003). For example, a study involving autistic children found that higher respiratory sinus arrhythmia reactivity, a potential marker for emotional regulation, affected the link between negative parenting and externalising behaviours (Baker et al. 2020), suggesting that emotional regulation may be involved in the behavioural difficulties exhibited in this population. As such, future research should examine potential bidirectional associations between parenting behaviours and child externalising problems, but also the role of emotion regulation.

Autistic children’s total difficulties increased over time, while our supplementary analysis of non-autistic children’s trajectories showed that they decreased under both high and low levels of harsh parenting. Moreover, irrespective of harsh parenting level non-autistic children’s scores remained in the normal range. While it is essential to acknowledge the sample size and gender composition difference between these groups, these findings could indicate different psychopathological pathways between autistic and non-autistic children. Previous research conducted with typically developing individuals and some autistic samples has demonstrated a positive correlation between harsh parenting and internalising problems in both cross-sectional (Hecker et al. 2016) and longitudinal (Rajyaguru et al. 2019; Speyer et al. 2022) studies, and in our supplementary analysis of the non-autistic sample this was also observed. Contrary to our hypothesis, this association in autistic individuals was not significant and, whilst this finding reflects similar studies looking at harsh parenting (Maljaars et al. 2014; McRae et al. 2018), it is not consistent with all studies (Dieleman et al. 2017; Lindsey et al. 2020). However, one must be cautious when drawing this conclusion due to over-representation of males compared to females in the autistic samples and low internal consistency of internalising problems in early sweeps. However, given females typically have higher levels of internalising problems compared to males, differences in the sex composition of autistic samples may have affected the observed results (Gutman & Codiroli McMaster, 2020). Nevertheless, it is important to elucidate why there may be differences in the drivers of internalising problems between autistic and non-autistic children.

This study had some notable limitations. Firstly, this is a correlational study and as such as we are unable to prove that maternal harsh parenting caused children to have more behavioural problems. Secondly, all measures were parent-reported and therefore could inflate correlations between them. Moreover, mothers may not want to disclose negative parenting behaviours due to social desirability. Thirdly, this study relied upon maternal harsh parenting, leaving out fathers’ harsh parenting, given unavailability of father harsh parenting data in the MCS. As such, future research should explore the joint role of mothers’ and fathers’ harsh parenting as it relates to child difficulties. Fourthly, the SDQ is made up of domains which can reflect core elements of autism, specifically peer problems. Fifthly, our harsh parenting and internalising problems scales had only adequate internal consistency and therefore the findings should be interpreted with caution.

While the study has several limitations, it also has considerable strengths. Our study has a large sample of autistic children which was drawn from a population sample rather than a clinical sample demonstrating how mental health problems unfold from a real-world perspective. Additionally, the use of a longitudinal design facilitated an investigation of how harsh parenting may relate to trajectories of emotional and behavioural difficulties across childhood. Our findings highlight that harsh parenting - smacking, shouting, and telling off - are likely to elevate autistic children’s behavioural problems, indicating that carers may benefit from reducing negative ways of responding to their child when they misbehave. Future research might explore these relationships considering fathers as well and in a sample with a greater number of autistic female children. Nevertheless, recognising this association and its potential outcomes empowers parents and caregivers to embrace beneficial parenting approaches that could ultimately ameliorate or prevent such challenges, thereby promoting the well-being of their autistic children. Additionally, identifying potential risk factors for mental health concerns in their children could ensure that these families access the best support available.

## Supplementary Information

Below is the link to the electronic supplementary material.

Supplementary material 1 (DOCX 171 kb)
