# Supplementary material for: Harsh Parenting and Trajectories of Emotional and Behavioural Difficulties in Autistic Children
Source: J Autism Dev Disord. 2023 Nov 4;54(12):4637–49. doi: 10.1007/s10803-023-06167-4 (PMC11549161; doi:10.1007/s10803-023-06167-4)
Supplement: Supplementary file 1 — Supplementary material 1 (DOCX 171 kb) [file 10803_2023_6167_MOESM1_ESM.docx]

**Supporting Information**

Harsh parenting and trajectories of emotional and behavioural difficulties in autistic children

Georgia Cronshaw, BSc.^1*^, Emily Midouhas, Ph.D.^1^

1 Department of Psychology and Human Development, IOE, UCL’s Faculty of Education and Society, London, UK

*Corresponding Author: georgia.cronshaw.19@ucl.ac.uk (Georgia Cronshaw)

This supplementary document contains the supporting information which includes the figures and tables for the supplementary analysis conducted in this study.

| **Table S1**  *Model Specification* | |
| --- | --- |
| *Within* | *Between* |
| *Y_ti_ = β_0t_ + β_1t_ AGE +ε_ti_* | *β_0t_ = γ_00_ +γ_01_Age^2^_i_ + γ_02_Gender_i_ + γ_03_Ethnicity_i_ + γ_04_Design_variables_i_ + γ_05_SES_i_ + γ_06_Maternal_Warmth_i_ + γ_07_Mothers_age_at_birth_i_ + γ_08_Maternal_Mental_Health_i_ + +γ_09_Maternal_Education_i_ + γ_010_* *harsh_parenting_i_ + γ_011_Age:harsh_parenting_i_ + μ_0i,_*  *β_1t_ = γ_10_ +γ_11_Age^2^_i_ + γ_12_Gender_i_ + γ_13_Ethnicity_i_ + γ_14_Design_variables_i_ + γ_15_SES_i_ + γ_16_Maternal_Warmth_i_ + γ_17_Mothers_age_at_birth_i_ + γ_18_Maternal_Mental_Health_i_ + γ_19_Maternal_Education_i_ + γ_110_* *harsh_parenting_i_ + γ_111_Age:harsh_parenting_i_ + μ_1i,_* |

**Fig. S1**


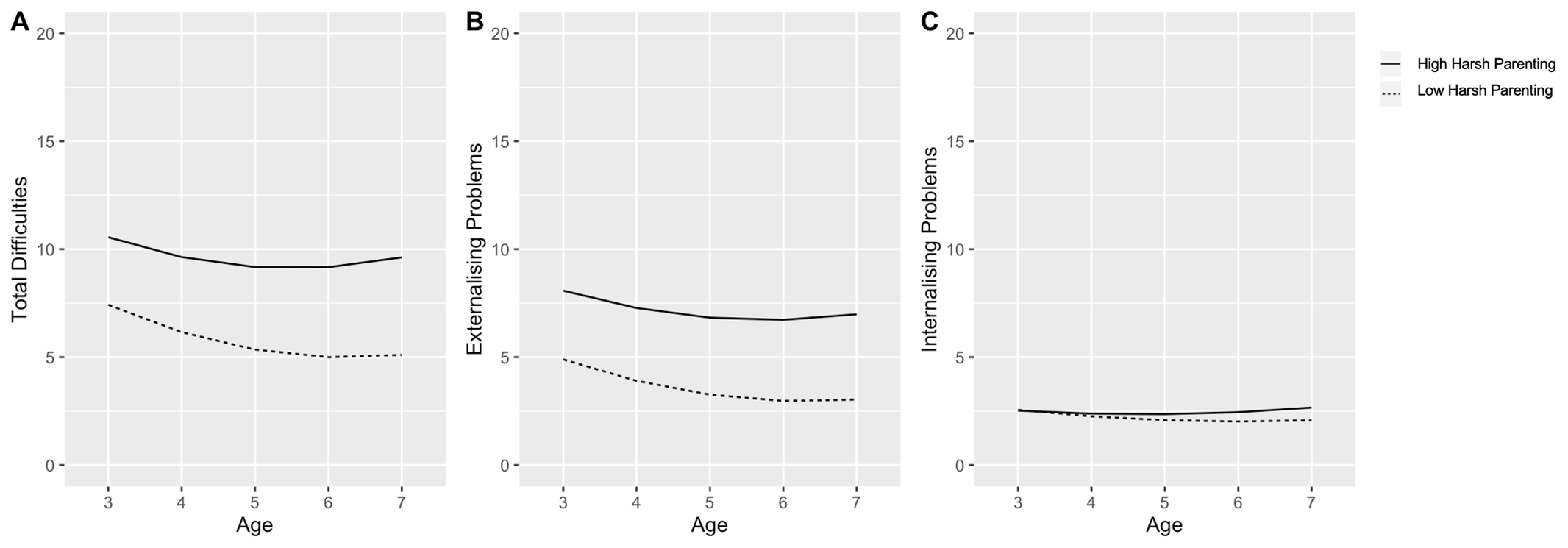
*Predicted trajectories for emotional and behavioural difficulties (n = 9879)*


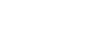

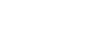


*Note.* a) total difficulties; b) externalising problems; and c) internalising problems from ages 3 to 7 in non-autistic children for both high and low levels of harsh parenting. High and low harsh parenting were calculated as the 90th and 10th percentiles respectively, with reference groups being used for categorical variables and the mean being used for continuous variables.

Descriptive Caption: Figure illustrates the trajectories of emotional and behavioural difficulties across different age groups

|  |  | **Table S2**  *Fixed and random effects estimates predicting total difficulties, internalising, and externalising problems (n = 9879)* | | | | | | | | | | | |  |
| --- | --- | --- | --- | --- | --- | --- | --- | --- | --- | --- | --- | --- | --- | --- |
|  | | | **Total Difficulties** | | |  | **Internalising Problems** | | |  | **Externalising Problems** | | | |
|  | | | $\beta$ | *SE* | *CI (95%)* |  | $\beta$ | *SE* | *CI (95%)* |  | $\beta$ | *SE* | *CI (95%)* | |
|  | | |  |  |  |  |  |  |  |  |  |  |  | |
| **Fixed Effects** | | |  |  |  |  |  |  |  |  |  |  |  | |
| Constant | | | 22.598*** | 0.347 | [-21.913-23.282] |  | 8.167*** | 0.187 | [7.800-8.533] |  | 14.341*** | 0.249 | [13.846-14.836] | |
| Harsh Parenting | | | 0.398*** | 0.014 | [0.371-0.425] |  | 0.029*** | 0.008 | [0.014-0.044] |  | 0.372*** | 0.010 | [0.353-0.390] | |
| Harsh Parenting X Age | | | 0.036*** | 0.007 | [0.023-0.049] |  | 0.016*** | 0.004 | [0.009-0.023] |  | 0.020*** | 0.004 | [0.011-0.029] | |
| Age | | | -0.623*** | 0.039 | [-0.701- -0.546] |  | -0.140*** | 0.022 | [-0.183- -0.097] |  | -0.489*** | 0.027 | [-0.541- -0.436] | |
| Age^2^ | | | 0.229*** | 0.009 | [0.212-0.246] |  | 0.059*** | 0.005 | [0.049-0.069] |  | 0.176*** | 0.006 | [0.164-0.187] | |
| Female | | | - 0.528*** | 0.064 | [-0.653- -0.403] |  | 0.046 | 0.034 | [-0.020-0.113] |  | -0.600*** | 0.046 | [-0.691- -0.509] | |
| *Poverty Status (Ref: SES advantaged)* | | |  |  |  |  |  |  |  |  |  |  |  | |
| Poverty Status disadvantaged | | | 0.973*** | 0.080 | [0.816-1.130 |  | 0.359*** | 0.043 | [0.275-0.443] |  | 0.629*** | 0.058 | [0.516-0.743] | |
| University Educated | | | -1.097*** | 0.079 | [-1.251- -0.942] |  | -0.219*** | 0.042 | [-0.301- -0.137] |  | -0.874*** | 0.057 | [-0.990- -0.761] | |
| Ethnicity Other (Ref: White) | | | 0.350* | 0.137 | [0.082-0.618] |  | 0.378*** | 0.072 | [0.236-0.520] |  | -0.051 | 0.098 | [-0.244-0.142] | |
| *Area stratum (Ref: England-advantaged)* | | |  |  |  |  |  |  |  |  |  |  |  | |
| England disadvantaged | | | 0.563*** | 0.088 | [0.390-0.736] |  | 0.206*** | 0.047 | [0.114-0.299] |  | 0.347*** | 0.064 | [0.221-0.473] | |
| England ethnic | | | 0.723*** | 0.166 | [0.398-1.048] |  | 0.427*** | 0.088 | [0.255-0.600] |  | 0.325** | 0.120 | [0.090-0.560] | |
| Scotland advantaged | | | -0.162 | 0.133 | [-0.423-0.099] |  | -0.084 | 0.071 | [-0.223-0.056] |  | -0.081 | 0.097 | [-0.271-0.110] | |
| Scotland disadvantaged | | | 0.202 | 0.143 | [-0.078-0.482] |  | 0.053 | 0076 | [-0.096-0.202] |  | 0.208 | 0.104 | [0.004-0.411] | |
| Wales advantaged | | | 0.064 | 0.153 | [-0.237-0.365] |  | -0.002 | 0.082 | [-0.162-0.158] |  | 0.083 | 0.112 | [-0.137-0.302] | |
| Wales disadvantaged | | | 0.453*** | 0.115 | [0.228-0.678] |  | 0.133* | 0.061 | [0.014-0.255] |  | 0.351*** | 0.083 | [0.188-0.515] | |
| Northern Ireland advantaged | | | -0.172 | 0.166 | [-0.496-0.152] |  | 0.047 | 0.088 | [-0.126-0.221] |  | -0.236 | 0.120 | [-0.472-0.000] | |
| Northern Ireland disadvantaged | | | 0.199 | 0.149 | [-0.092-0.490] |  | 0.107 | 0.079 | [-0.048-0.262] |  | 0.077 | 0.108 | [-0.135-0.289] | |
| Maternal Warmth | | | -0.246*** | 0.005 | [-0.256- -0.236] |  | -0.072*** | 0.003 | [-0.077- -0.067] |  | -0.172*** | 0.004 | [-0.179- -0.164] | |
| Maternal Mental Health | | | -0.185*** | 0.008 | [-0.120- -0.170] |  | -0.101*** | 0.004 | [-0.109- -0.093] |  | -0.089*** | 0.005 | [-0.099- -0.079] | |
| Maternal Age at Birth | | | -0.060*** | 0.006 | [-0.072- -0.048] |  | 0.016*** | 0.003 | [-0.022- -0.010] |  | -0.042*** | 0.004 | [-0.050- -0.033] | |
|  | | |  |  |  |  |  |  |  |  |  |  |  | |
| **Random Effects** | | |  |  |  |  |  |  |  |  |  |  |  | |
| Level 2 (child) | | |  |  |  |  |  |  |  |  |  |  |  | |
| Between-child intercept variance | | | 6.74 |  |  |  | 1.78 |  |  |  | 3.78 |  |  | |
| Between-child slope variance | | | 0.44 |  |  |  | 0.13 |  |  |  | 0.22 |  |  | |
| Between-child intercept/slope variance covariance | | | 0.42 |  |  |  | 0.11 |  |  |  | 0.12 |  |  | |
| Level 1 (occasion) | | |  |  |  |  |  |  |  |  |  |  |  | |
| Residual variance | | | 6.22 |  |  |  | 2.22 |  |  |  | 3.21 |  |  | |
|  | | |  |  |  |  |  |  |  |  |  |  |  | |
| *Note.* $\beta$ = coefficient; SE = Standard Error; CI = 95% confidence intervals. * *p* < .05. ** *p* < .01. ****p* < .001 | | | | | | | | | | | | | | |
